# Ink-Jet Printing of Micro-Electro-Mechanical Systems (MEMS)

**DOI:** 10.3390/mi8060194

**Published:** 2017-06-21

**Authors:** Gih-Keong Lau, Milan Shrestha

**Affiliations:** 1 School of Mechanical and Aerospace Engineering, Nanyang Technological University, Singapore 639798, Singapore; MILAN001@e.ntu.edu.sg; 2 Singapore Center of 3D Printing, Nanyang Technological University, Singapore 639798, Singapore

**Keywords:** inkjet printing, micro-electro-mechanical systems, sensors, actuators

## Abstract

Beyond printing text on paper, inkjet printing methods have recently been applied to print passive electrical and optical microparts, such as conductors, resistors, solder bumps and polymeric micro lenses. They are also useful to print micro-electro-mechanical systems (MEMS) as sub-millimeter sensor and actuator arrays, such as multifunctional skins applicable to robotic application and ambient monitoring. This paper presents the latest review of a few successful cases of printable MEMS devices. This review shows that inkjet printing is good for printing two-dimensional or surface MEMS devices from a small unit to an array over a large area. In the future, three-dimensional printing of multi-materials, from metal, plastic, to ceramic, will open the possibility of realizing more variety and function of a large-areal MEMS array, for a mobile electro-mechanical systems.

## 1. Introduction

Micro-electro-mechanical systems (MEMS) are sensors or actuators of micro-meter to sub-millimeter size [1,2]. MEMS sensors can convert an input of a mechanical measurand into an electrical output signal. For example, MEMS accelerometers and gyroscopes [1,3] are used in a smartphone to sense the orientation/rotation of a display screen. MEMS actuators can convert an electrical input into a controllable output of mechanical response. For example, a micro-mirror array is used to switch pixels of the light projection display (i.e., Texas Digital Light Processor) [1,2,3].

Sensing principles of MEMS include one of the following [1,2,3]: (1) capacitive sensing to measure an electrode displacement with the output of capacitance change; (2) piezo-resistive sensing to measure a stress or strain in a deformable element with the output of a resistance change; (3) piezoelectric sensing to measure the stress in a piezoelectric ceramic with the output of electric charge. The actuation principles include [1,2]: (1) electrostatic attraction to close the air gap between a movable electrode and a stationery one; (2) converse piezoelectric effect to induce a strain in the piezoelectric ceramic subjected to an electric field; (3) solid thermal expansion as induced by resistive heating.

MEMS consist of moving micro-structural elements, integrated electrodes and even active material under the control of integrated microelectronics. MEMS are mostly fabricated on silicon substrate, which is the choice for fabricating integrated microelectronics. Yet, silicon micro-machining is expensive and tedious in steps, involving photolithography, material addition and material subtraction. Silicon micro-machining of MEMS devices is done in batch and often has a low yield at the initial trials. Successful making of MEMS devices required many runs and optimization of established micro-fabrication processes. Alternatively, inkjet printing methods or additive manufacturing can help rapid prototyping of MEMS devices [4,5,6,7], albeit larger at a sub-millimeter size.

Inkjet printing methods are not just limited to printing of color inks on paper [4], but they also apply to printing of functional devices [6,7]. The inkjet printing technology has a print head to eject and direct liquid droplets at a precise location on a paper or flexible substrate. There are two modes of inkjet printing [4,7], namely a continuous mode for industrial marking or product labeling and an on-demand mode for home/office-use inkjet printers. For example, a conductor can be printed from conductive colloid [5,6] or molten solder [5,7]. Inkjet printing methods readily make passive electrical components, like a planar conductor, a planar inductor (coil), even a coplanar air capacitor. Yet, printing of moving elements, such as a suspended spring, is more challenging due to the need for printing multi-materials, into the over-hanged micro-structure with support on a sacrificial layer.

Inkjet printing does not make a MEMS device as dimensionally precise as conventional methods based on photolithography and silicon micro-machining processes. Lateral resolution of printed pattern is not as high as that formed by lithography. In addition, thickness control of the printed layer is not better than those deposited by the physical or chemical vapor deposition methods. However, printing is inexpensive and effective to make arrays of MEMS or sub-millimeter sensors and actuators distributed over a large area. For example, the inkjet printing method has successfully been used to print: (1) a microlens array as the areal light diffuser in the light guide plate of a display [8]; (2) air-gap MEMS switches on plastic lamination for RF power transmission [9]. Recently, the printing method even makes distributed and flexible sensors and actuators for the application to robotic skin or environmental monitoring. One of the impressive examples is a large-area electro-adhesive pad as grippers [10].

Calvert (2001) [5] presented a review of inkjet printing of electrical and optical micro-parts, mostly electrically passive. Wallace et al. (2007) [7] reviewed the use of inkjet printing as the manufacturing tool for passive components such as solder bumps and microlenses. Bessonov and Kirikova (2015) [11] reviewed flexible sensors based on roll-to-roll printing or screen printing. Yet, few have reported the inkjet printing of MEMS actuators or sensors due to complexity in the making of moving elements. In the following sections of this paper, we shall review the successful and exciting development of printable MEMS devices as sensors and actuators, in addition to printing methods and materials.

## 2. Printing Methods

**Figure 1:**
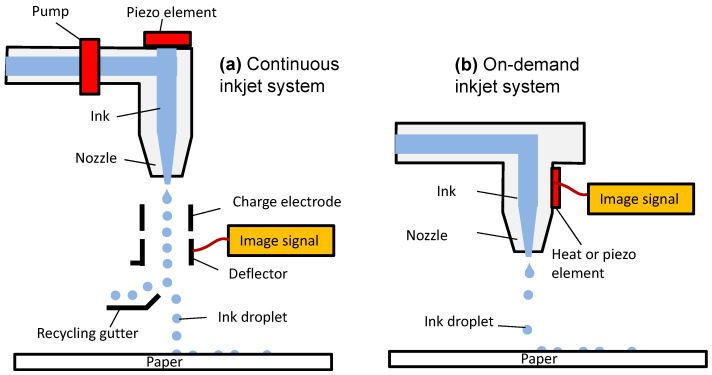
Schematics showing: (**a**) a continuous inkjet printer; (**b**) an on-demand inkjet printer. Redrawn from Ref. [12].

Figure 1 shows a basic print head consisting of a piezoelectric diaphragm above or on the side of an ink channel right above the nozzle [4,12]. Dynamic deflection of the pulsed-voltage activated piezoelectric diaphragm can generate a pressure wave to eject the ink out of the nozzle, squeezing out a liquid jet that breaks into droplets due to liquid’s surface tension. A continuous mode [4,7] of ink printing has a faster rate of 0.5-μL droplets generation for 80 kHz–100 kHz. A deflection plate is electrically controllable to aim the droplets on to a substrate. The excess droplets are recirculated from the gutter. A demand-mode printing system [4,7] has a slower ejection rate (of up to 30 kHz) of smaller ink droplets (2 pL–500 pL) [7], without the recirculation system as for the continuous system. Hence, the demand mode printing system has a simple design and wastes less ink. Some brand names of on-demand inkjet material printers for research and development use are: Dimatix material printer (DMP-2850) from Fujifilm USA and Omnijet 300 from Unijet Korea.

The normal print head can process inks with a viscosity up to 30 cP. Water or solvent-based ink has a viscosity below 5 cP [13]. Yet, molten polymer or mixture could have a much higher viscosity. A special print head was recently developed to process high-viscosity (of up to 500 cP) fluid [14].

Liquid solvent is commonly used to dissolve solid resin or suspend solid nanoparticles to make the ink have the right viscosity (2 cP–100 cP) for inkjet printing. The ideal solvent should also have a low evaporation rate to prevent fast drying from causing the clogging of the nozzle. The substrate for printing is usually heated at 100 ${}^{\circ }$C–300 ${}^{\circ }$C to improve the print quality [6]. Flash evaporation occurs to dry up the liquid in a printed droplet upon its contact on the hot substrate. This eliminates the smudging problem and allows rapid printing of multiple layers. The drying process depends strongly on the selected solvent. Smudging of liquid ink droplets can reduce the print quality and resolution.

Lateral resolution of a printed line depends on the ink droplet size and the ink’s spread on a substrate. Typically, a demand-mode print head ejects a smaller droplet (20 μm–100 μm diameter). In comparison, a continuous-mode print head produces a larger droplet of up to 0.5 mm in diameter [15]. A resolution of greater than 20 μm is possible by high precision inkjet printing on a proper substrate, but an average resolution of a few 100 μm is more widely achievable [6]. The omniphobic surface on a substrate is desirable for high resolution printing [10]. Too much ink spread upon wetting on the substrate will reduce the resolution; whereas, no spread of ink on a phobic substrate does no help to form a continuous line from separate ink droplets.

A common substrate for inkjet printing is paper. Yet, a paper is not necessarily the best substrate for high-resolution printing of special inks. Conductive inks, like silver ink or carbon ink, are often printed on plastic, glass or silicon wafer where the printed ink droplets do not spread too much for good lateral resolution. The plastic substrate is preferred for high flexibility. Recently, omniphobic paper with fluoroalkylated coating enabled high-resolution printing of silver or carbon inks with a lateral resolution greater than 80 μm. A commercial paper (e.g., pe:smart paper type 2 from Felix Schoeller) for printed electronics has a nanoporous coating made of oxide film and a 3.3-nm roughness to improve inkjet printing [16].

**Figure 2:**
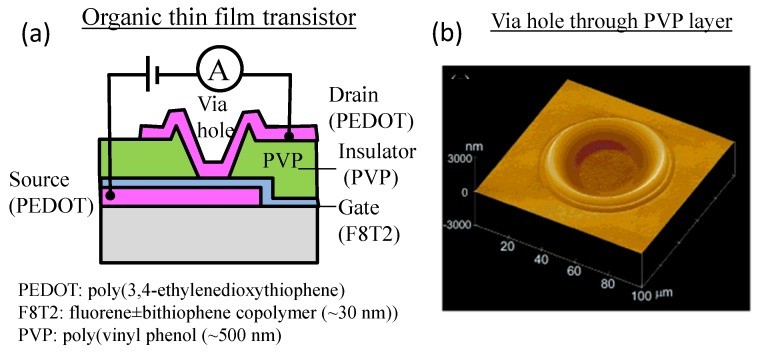
“Printing” of solvent droplets to create a via hole through a polymeric insulator. (**a**) Organic thin film transistor; (**b**) via hole through the PVP layer. Reproduced and adapted with permission [17]. Copyright 2001, John Wiley and Sons.

Besides adding material, printing of solvent can help remove material. Printing of solvent helps create a via hole through a polymeric insulator layer between the printed polymeric electrodes in an organic thin film transistors (see Figure 2). For example, Kawase et al. [17] show that a droplet of solvent can locally dissolve the insulator poly(vinyl phenol) ((PVP) of 500 nm thick), which was spin coated from the solution of xylene and isopropanol (IPA). The printed solvent for material removal dries within a second, with re-solidification of the insulator from the solution. The dissolved solid PVP insulator is reflowed towards the edge of solvent puddle. Repeating the solvent drop and drying at the same location creates a crater-like via hole through the insulator layer.

## 3. Printable Materials

This section presents a review of various materials suitable for inkjet printing or modified for printing.

### 3.1. Conductive Materials

Electrical conductors are the signal path, as well as the power line to MEMS. They make electrodes, heaters or passive micro-structures. Printing of conductors is based on either (1) conductive ink or (2) molten solders.

Conductive ink [6,18] is a colloid consisting of conductive nano-particles dispensed in liquid solvent. For example, 5–7 nm gold or silver nano-particles were well dispensed in α-terpineol to make conductive ink for printing [6]. A commercial silver nanoparticle dispersion in tetradecane (NPS-JL, Harima Chemicals Inc., Tokyo, Japan) has a 55 wt % solid content of silver nanoparticles (of diameter from 5 nm–12 nm) [19]. Use of nano-particles less than 50 nm in diameter helps to avoid aggregation and sedimentation encountered by larger nanoparticles [5]. Viscosity of the ink increases with the solid loading of nano-particles. Higher loading concentration of the solid nanoparticles may cause the clogging of nozzles. Dilution with solvent can reduce the viscosity of the nanoparticle dispersion. Substrate heating is necessary to evaporate the liquid and keep only the solid conductive material. Sintering at an elevated temperature for an extended duration (e.g., 300 ${}^{\circ }$C for minutes [6]) is necessary to improve the conductivity of the printed conductor.

Recently-developed inks based on metal nanoparticles can be printed and sintered at a lower temperature. Metal nanoparticles exhibit low melting temperature due to the thermodynamic size effect [18]; for example, 3-nm Au nanoparticles start to melt below 150 ${}^{\circ }$C, i.e., considerably lower than the bulk melting temperature (1064 ${}^{\circ }$C). An example of silver ink with a low sintering temperature of 150 ${}^{\circ }$C for 1 h is based on less than 50-nm diameter silver nanoparticles dispersed in triethylene glycol monomethyl ether (silver dispersion 736465, Sigma Aldrich, Singapore) [20]. In addition, it is noted that the new conductive ink can be printed and sintered to achieve as much as 70% of the bulk metal’s conductivity [21].

Printing a solution of metal pre-cursor, followed by a conversion, also makes a metal line [5]. Silver metalorganic ink [22] converts to metal below 300 ${}^{\circ }$C. The firing step and heat treatment are necessary to make the multilayer precursor converted metal line with low resistance for photovoltaic cells. The addition of bismuth to the precursor improves the interlayer adhesion.

Solders make the electrical interconnect and bumps for electronic packaging. They are categorized into (1) lead-containing solder or (2) lead-free solder due to the concern over health hazards. An example of lead-containing solder is eutectic tin-lead (63% Sn and 37% Pb); while an example of lead-free solder is SAC (96.5% Sn, 3% Ag, 0.5% Cu) [7]. The piezoelectric inkjet printer was successfully used to generate jets and droplets from molten solder. The SolderJet [7] produces solder droplets with a size ranging from 20 μm–125 μm at a rate of 1000 droplets per second.

### 3.2. Insulator Materials

The insulator layer is needed to make a passivation coating over or under an electrode. It also makes a dielectric layer in a capacitor. Few printed dielectric material can sustain a high electrical breakdown field due to air voids and material inhomogeneity. The solution of dielectric pre-polymer or pre-cursors, which was developed for spin coating, can be diluted for printing, provided they do not clog the nozzle of the print head. An example of a printable insulator [6] is based on polyketone resin dissolved by 12% by weight in a solvent mixture of 50% ethyl lactate and 50% α-terpineol. The 10-layer printed polyketone (with a total thickness of 40 μm) can withstand 700 V (i.e., 700 V/40 μm = 17.5 MV/m). Other printable soft dielectrics are silicone elastomer that comes in two-part pre-prepolymer liquids, which were mixed and diluted for spray coating [23].

Other traditional liquid dielectric, such as spin-on-glass (Filmtronic 500 FX, Butler, PA, USA) [6], may cause the clogging of the nozzles, though they can be diluted to have the viscosity and solid content suitable for printing. To have a very thin and high-performance dielectric layer, a traditional deposition method like spin coating is still preferred to the printing method. For example, poly-4-vinylphenol (PVP) [21] was spin-coated and cross-linked at 220${}^{\circ }$ to make a 0.3 μm-thick dielectric layer over a gated electrode of an electrostatic field-effect switch.

### 3.3. Sacrificial Materials

A sacrificial layer is needed to temporarily support a structural layer printed. Removal of the sacrificial layer will release the structural layer for deformable movement. There are not many choices of sacrificial material due to the need to withstand higher substrate heating and to be dissolvable by solvent. Poly-methyl-methacrylate (PMMA) was chosen as the sacrificial material to temporarily support a metallic micro-cantilever because it can withstand 300${}^{\circ }$ of heating for sintering of the printed metallic inks. A PMMA layer is prepared by spin coating or draw casting of a solution containing PMMA solid (50% by weight) [6] or liquid pre-polymers of crosslinkable PMMA. Solvent removal by heating and evaporation from the liquid film yield a solid layer of PMMA. Complete removal of the PMMA sacrificial layer is done by immersion in an acetone bath with light sonication. Selective removal [21] is done by printing acetone over the PMMA patch, for example at the anchor, where metal inks are to be printed. Yet, selective removal of PMMA by printed acetone yields a crater-like hole for metallic anchoring [21].

### 3.4. Piezoelectric Materials

Piezoelectric materials constitute an active part in a sensor or actuator. They are either ceramic or polymer. Piezoelectric ceramics include zinc oxides, lead-zirconate-titanate (PZT) and barium titanate (BaTiO${}_{3}$). A few piezoelectric polymers are poly(vinylidene fluoride) (PVDF) and its copolymers with trifluoroethylene (TrFE) and tetrafluoroethylene (TFE). Piezoelectric polymers shows much less piezoelectric effect than the piezoelectric ceramics, but they are solution processable and ready for inkjet printing. PVDF-TrFE film can manifest the piezoelectric effect upon electrical poling [24], without the need for stretching as for PVDF film. In addition, PVDF-TrFE has a higher crystallinity than PVDF does, and thus, the former shows a higher remanent polarization being thermally more stable (up to 100 ${}^{\circ }$C) [25].

Piezoelectric ceramic can be prepared from either a green paste of oxide particles [26,27] or a sol-gel liquid [20,28,29]. Green paste is screen printed on a substrate or molded into a bulk piece alone. Sol-gel is solvent cast and evaporated onto a substrate. They are subsequently sintered and poled before turning into the active piezoelectric layer [26,28,30]. Dicing is commonly used to cut or engrave piezoelectric ceramic in patterns, but risks breaking the brittle ceramic. Recently, printing technology provides a facile way to pattern the slurry [31] of piezoelectric particles or sol-gel before they are sintered into piezoelectric ceramic.

PVDF and its copolymers with TrFE are highly soluble in polar organic solvent, such as dimethylformamide (DMF) [32], cyclopentanone [19], dimethylacetamide (DMAc), *N*-methyl-2-pyrrolidone (NMP) [33] and methyl ethyl ketone (MEK) [34]. These piezoelectric polymers can be processed into thin films by solution casting and annealing close to the crystallization temperature. Hence, the solution containing the piezoelectric polymer resin is inkjet printable. For example, a 2 wt % solution of PVDF in cyclopentanone was inkjet printed on to a metalized plastic substrate [19].

### 3.5. Other Functional Materials

Functional materials make a strain gauge, a thermistor or an active layer of a field-effect transistor (FET) [11]. They can make skin-inspired FET sensors for pressure and temperature detection. The charge density carrier of the functional material changes in response to the interaction with stimuli. Some useful functional materials includes: PEDOT:PSS (poly(3,4-ethylenedioxythiophene polystyrene sulfonate), graphene, carbon nanotube, carbon black or their elastomeric composites. They can be patterned by means of inkjet printing or roll-to-roll printing for mass production. Some of these functional materials can be inkjet printed for rapid prototyping of sensors.

## 4. Printed MEMS Devices

This sections presents a review of the MEMS devices made by inkjet printing methods or a hybrid of other methods.

### 4.1. Microlens

**Figure 3:**
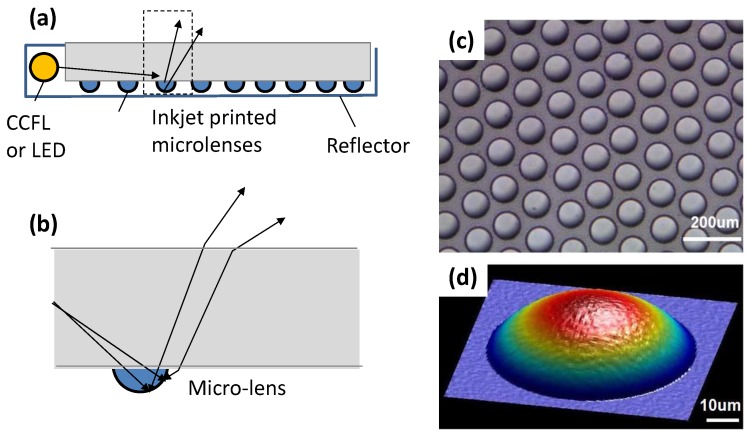
Microlens array on a seven-inch light guide plate, together with white reflector, for display: (**a**,**b**) schematics of light guide plate with the microlens array; (**c**,**d**) optical micrograph and topology of microlenses. [2009] IEEE. Reprinted, with permission, from [8].

The microlens is useful to couple light between the source and detector by light beam shaping. According to Cox et al. [35], 125 μm-diameter microlenses were printed onto the tips of multimode optical fibers to increase their acceptance angles by at least a factor of three. In addition, an array of inkjet-printed microlenses (see Figure 3) is useful as light deflectors, converting side lighting, such as a cold cathode fluorescent lamp (CCFL) or light emitting diode (LED), into planar back-lighting for a liquid crystal display (LCD) [8].

Inkjet printing reduces the time for microlens prototyping, eliminating the need for mold making. Printable materials for the microlens are UV-curable liquid pre-polymer like SU8 epoxy [36] and modified poly(methyl methacrylate) (PMMA) [8]. They can be printed as droplets on the substrate like an acrylic or glass plate. The contact angle between the ink droplet depends on the ink viscosity and substrate surface treatment. Perfluorine hard coating on PMMA makes the hydrophobic surface good for creating a nearly spherical droplet. After being printed, the pre-polymer droplets are UV cured into solid microlenses. A typical microlens in the array for the light guide plate has a diameter of 50 μm, a sag of 5 μm and a 65 μm radius of curvature [8].

### 4.2. Solder Bump

As electrical interconnects to the external circuitry, solder bumps are deposited on pads of an integrated circuit (IC) chip. This method of electrical interconnect is commonly known as a flip-chip. In mass production, screen printing of solder paste or electrode plating through a sacrificial mask is commonly used to deposit solder material onto the pads of an IC chip. A reflow process at the melting temperature is required to make the deposited solder material into bumps or balls. Alternatively, inkjet printing provides a facile method to form solder bumps, saving the process steps for temporary masking and reflow.

**Figure 4:**
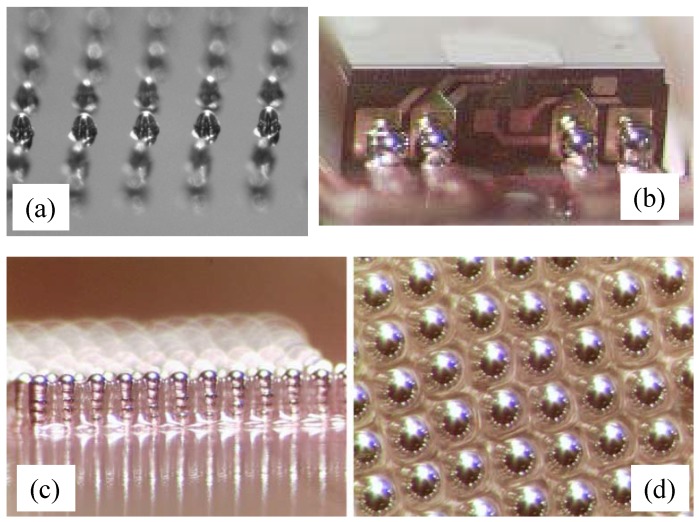
Solder bump printed from molten solder : (**a**) solder bumps on a chip; (**b**) solder bumps for a read-write head; (**c**,**d**) solder towers in the angled and top views, respectively. Redrawn from Ref. [37] with permission of Dr. David B. Wallace, MicroFab Technologies, Inc.

SolderJet [7,37] can print solder bumps of 25 μm–100 μm in diameter. Solder material is molten at 200 ${}^{\circ }$C–300 ${}^{\circ }$C. Printing of the molten solder happens in inert gas, which prevents oxidation of the solder droplet. Figure 4 shows an array of 100 μm in diameter solder bumps. Such printed solder bumps can also make the electrical interconnect at the corner between the vertical signal pad of a read-write head and the plane of a flexible circuit. In addition to making solder bumps, the printing method is also applicable to fabricate vertical vias across multilayer printed circuit board. Multiple printing of molten solder on the same spot creates a solder tower, for example 240 μm-high towers with printed polymer fillers in the 150-μm pitch gaps. The polymer fillers act as a mold to keep the shape of a solder tower during the reflow process.

### 4.3. Conductor

**Figure 5:**
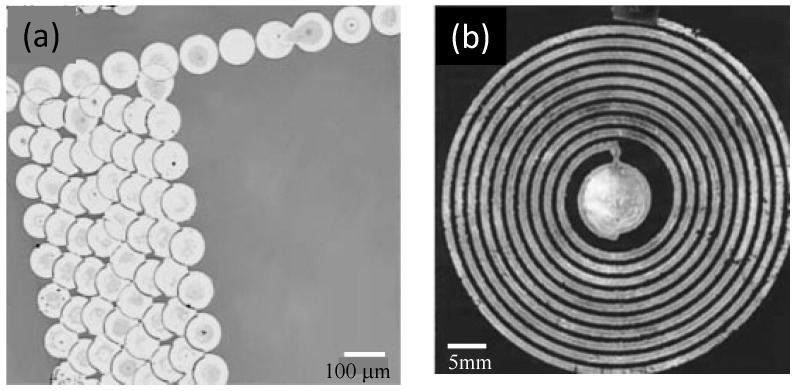
Conductors printed from silver nanoparticle ink: (**a**) printed ink droplets; (**b**) printed coil [6]. Reproduced with permission [6]. Copyright 2002, IEEE.

Inkjet printing of metallic nanoparticle ink can deposit a conductive dot on a heated substrate, for example an 80–100-μm diameter dot on a 300 ${}^{\circ }$C substrate of polyimide film. Tracing the same path by printing and drying of ink droplets yields a thicker multi-layer ink line on the substrate. Multiple overlaps of printed droplets ensure the continuity of the printed conductive line or pad, while multiple tracings make thicker multilayers. If the substrate heating is lower (<150 ${}^{\circ }$C), the multiple droplets of ink will reflow and congregate on the substrate before complete solvent evaporation. Such printed metallic lines can make a resonant LC circuit for a radio-frequency identifier (RFID) tag. For example, Figure 5 shows a spiral silver coil [6] of 10 turns and 1.3 mm wide printed out of five layers of silver nanoparticle ink with a layer thickness of 100 nm on a substrate of polyimide film. Such a metallic coil together with air capacitance makes an LC circuit to absorb radio frequency signal at 150 MHz.

### 4.4. Resistance Temperature Detector

A resistance temperature detector is a thermometer, which has a resistance change linearly proportional to the temperature change. Platinum is a common material for making an resistance temperature detector (RTD) due to its high temperature coefficient of resistance (TCR). Recently, research [16] shows that an inkjet-printed silver line with parylene coating on a paper exhibits a good linearity with a TCR of 0.0011/${}^{\circ }$C. This silver resistor is printed from silver nanoparticle ink (DGP 40LT-15C, Anapro, São Paulo, Brazil). It has a line width ranging from 140 μm–340 μm and a meandered shape of extended length to increase the total resistance. The parylene coating serves as a barrier or passivation coating against humidity and oxygen from adversely affecting the silver’s linearity of resistance change with temperature.

### 4.5. Strain Gauge

A strain gauge is a resistor that changes its electrical resistance when subjected to a mechanical strain. It is usually printed on a flexible substrate to make a sensor for deflection. The shape of a strain gauge varies, be it a straight line, a U shape or a meandered shape for extended length.

**Figure 6:**
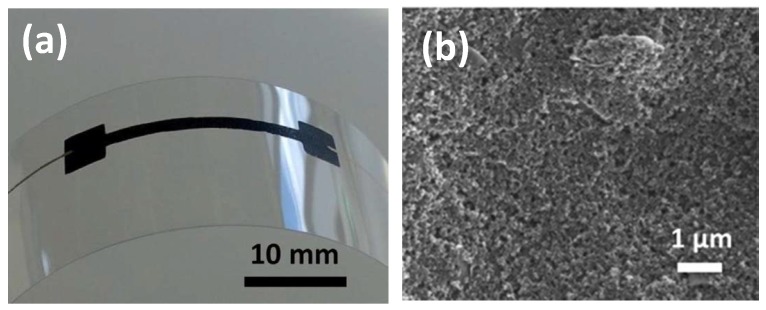
Printed graphite strain gauge on a plastic film: : (**a**) flexing of the gauge on a plastic film; (**b**) morphology of graphite coating. Reprinted from [38], Copyright (2014), with permission from Elsevier.

Various printable materials show a piezoresistive effect [11]. They are conductive polymers, metal nanoparticles and carbon nano-materials, elastomeric composite loaded with conductive nano fillers. Graphite [38] is reported with a high gauge factor of 19.3 when deposited by screen printing on a polymeric substrate. Figure 6 shows a graphite line of 20 mm–30 mm long, 0.5 mm–1.5 mm wide and an average thickness of 6.6 ± 0.4 μm, screen printed on a polyethylene naphthalate (PEN) film. Such a graphite strain gauge on plastic film can flex and show a linear resistance change over a small strain. The graphite ink for printing is a reformulation and dilution of graphite paste of high viscosity (26-8203, Sun Chemical, Parsippany-Troy Hills, NJ, USA) [38].

In comparison, inkjet-printed carbon nanotubes [39] were reported with a lower gauge factor ranging between one and two. This CNT ink is a dispersion of multi-walled carbon nanotubes (0.02 wt %) in dichlorobenzene with improved stability by surfactant [39]. A printed carbon-ink strain gauge (Methode 3801, Methode Electronics, Inc., Richardson, TX, USA) on a omniphobic paper (Model No. 702-442, Canson Vellum, France) can sense the tip deflection of the paper cantilever [10].

### 4.6. Heat Actuator

**Figure 7:**
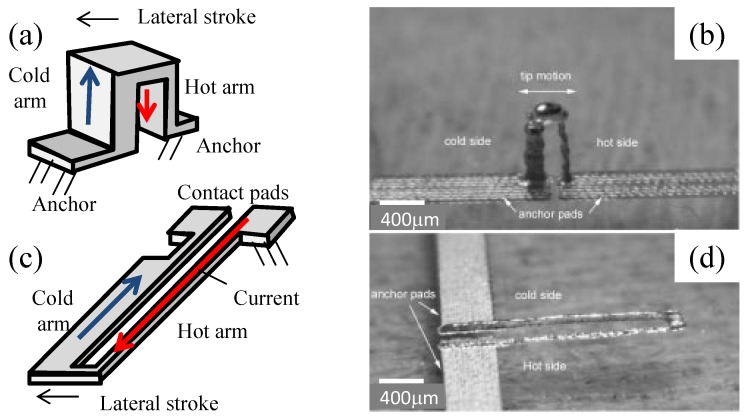
Heat actuators printed from silver nanoparticle inks: (**a**,**b**) a schematic and a prototype of a vertical heat actuator; (**c**,**d**) a schematic and a prototype for a pair of lateral heat actuators. Images (b) and (d) reproduced with permission [6]. Copyright 2002, IEEE.

A heat actuator is a U-shaped monolithic resistor subjected to differential thermal expansion [40,41,42]. The U-shaped resistor has a hot arm connected with a cold arm (see Figure 7). The hot arm has a smaller cross-section and a higher resistance; it generates more resistive heating when a current passes through the U-shaped resistor. Due to the differential resistive heating, the hot arm expands axially more than the cold arm. Hence, this creates a lateral motion towards the side of the cold arm.

Printing of multiple conductive layers can make a heat actuator [6]. A 1 mm-tall vertical heat actuator is printed out of 400 layers of silver nanoparticle inks on a glass slide (see Figure 7a,b). The printing was carried out at the substrate heating of 300 ${}^{\circ }$C and took 30 min. The printed pillars of hot and cold arms were joined at the top due to the mushrooming of the liquid top without the need for a sacrificial layer.

A planar heat actuator [6] (see Figure 7c,d) was obtained by printing multiple layers of silver nanoparticle ink on a glass slide and a PMMA sacrificial patch. This planar resistor has a hot arm made of 40 silver layers and a cold arm mode of 120 silver layers. The printed planar resistor is released upon the removal of the sacrificial patch. The difference in height results in a resistance difference between the hot and cold arms given the same line width. A 5-V activation of the planar heat actuator produces a 200-μm tip stroke.

### 4.7. Air-Gap Electrostatic Switch

**Figure 8:**
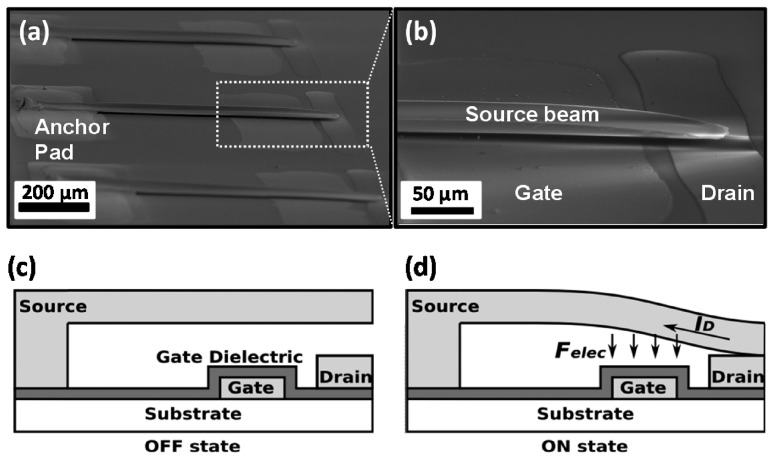
An electrostatic switch printed on a Si wafer: (**a**,**b**) scanning electron micro-graphs; (**c**,**d**) schematics showing the working of the switch. Reprinted with permission from [21]. Copyright 2013, American Chemical Society.

An air-gap electrostatic switch consists of a deformable electrode (i.e., a metallic micro-cantilever or a rotor) suspended at an air gap above a stationary electrode (i.e., stator) on a substrate. Insulation of the ground electrode (stator) is necessary to prevent a permanent stiction between the two electrodes and allows reversible switching. Park et al. [21] reported a printed relay micro-switch (see Figure 8) made of a silver micro-cantilever of 650 μm long, 90 μm wide and 2.25 μm thick, suspended at a 1.65-μm air gap above the passivated silver electrode on a substrate of an oxidized Si wafer. The passivation coating is 0.3 μm-thick poly-4-vinylphenol (PVP) spin coated and cross-linked at 220 ${}^{\circ }$C. The release of the micro-cantilever is obtained by selective removal of a patch of a 1.8 μm-thick PMMA sacrificial layer by using printed acetone puddles.

Such a printed relay switch shows a low on-state resistance (10 Ω) and a very low off-state leakage. Activation of the switch by applying a voltage difference between the source electrode (micro-cantilever) and the gate electrode will cause electrode deformation to make electrical contact with the drain electrode. Removal of the applied voltage will open and break the circuit.

**Figure 9:**
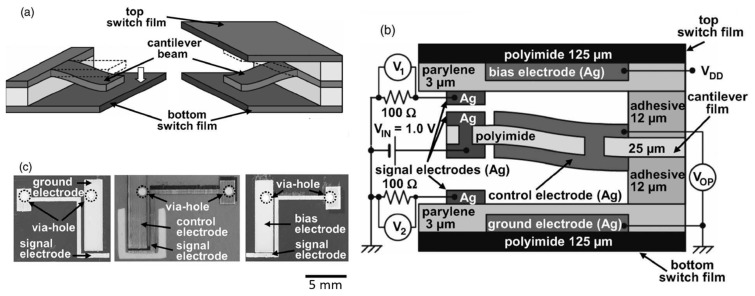
An electrostatic switch printed on a flexible polyimide film: (**a**,**b**) schematic showing the switch design; (**c**) optical micrographs (top view) showing each layer. Reprinted from [43], with the permission of AIP Publishing

A relay switch can also be printed on a flexible substrate like a polyimide film. Yet, a simple construction of relay switch on a flexible film is prone to false operation due to the external pressure disturbance. To make a reliable switch, Yokota et al. [43] proposed and developed a three-layer stack enclosing a deformable cantilever (see Figure 9). Such a device is made by laminating three polyimide films with inkjet-printed electrodes. The three films are a top switch film, a cantilever film and a bottom switch film. Their spacing of 12 μm is controlled by stacking with two adhesive spacers. The cantilever film was laser cut or mechanically cut to form a cantilever of 6 mm × 4.5 mm. The electrode on each film is printed from silver nanoparticle inks with a 80 ${}^{\circ }$ substrate heating and a 180 ${}^{\circ }$C sintering for 1 h. The electrodes are passivated by a 3 μm-thick parylene layer by chemical vapor deposition.

**Figure 10:**
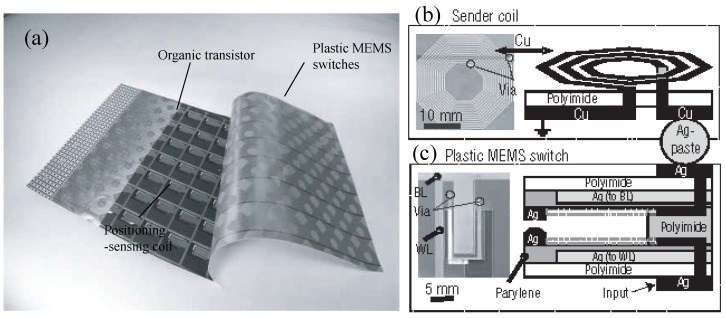
Plastic MEMS switches for RF power transmission: (**a**) photograph of the switch sheet; (**b**) photograph and schematic of a sender coil; (**c**) photograph and schematic of a plastic MEMS switch. Reprinted by permission from Macmillan Publishers Ltd: Nature [9], copyright 2007

Similarly, Sekitani et al., [9] developed a printed plastic MEMS switch sheet (see Figure 10) for turning on or off a sheet of sender coil arrays for a wireless power transmission system. The power transmission electrode of the plastic MEMS switch is connected to the alternating current (AC) power source operating at a frequency of 13.56 MHz. Once the MEMS switch is turn on, current starts flowing through the sender coil and thus generates a magnetic field. In turn, the receiver coil is subjected to a current flow as induced by the magnetic field. The receiver coil spaced at a distance from the sender coil can transmit an AC power as much as 2 W. This MEMS switch consists of three polyimide films stacked up, namely a top electrode film, a spacer film and a bottom electrode film, all with inkjet-printed silver electrodes. A printed plastic electronic and screen-printed positioning coil are used to control the switching.

### 4.8. Electro-Adhesion Pad

Parallel to an electromagnet, an electro-adhesion pad [10,44,45,46,47,48,49] provides an electrostatic means for robotic grippers to adhere to an object, either an insulator or a conductor. The electro-adhesion pad is basically a co-planar capacitor with a pair of interdigital electrodes on the surface of a dielectric layer [44,45,46]. Applying a high voltage between the interdigital electrodes of opposite polarity induces electrostatic fringe fields, which in turn induce adhesive force to an adjacent object. Removal of high voltage can switch off the adhesion to release an object. Such electro-adhesion pads have been used as grippers and wall-climbing robots, replacing air suction cups [10,44,47,49].

**Figure 11:**
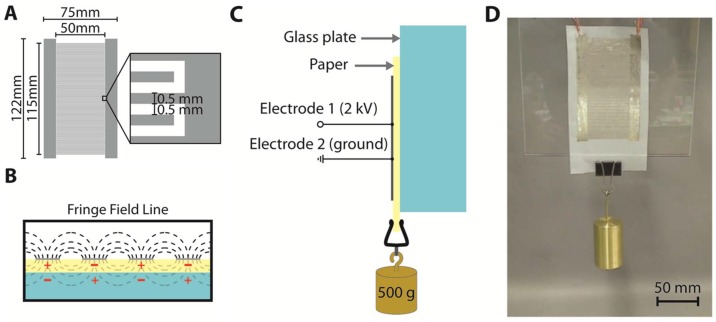
Electroadhesion based on printed silver interdigital electrodes on omniphobic Canson tracing paper : (**A**) design of interdigital electrodes; (**B**) fringe field induction around coplanar electrode; (**C**) test setup for electroadhesion; (**D**) 2-kV activated electro-adhesion in action, lifting a 500-gram weight. Reproduced with permission [10]. Copyright 2014, John Wiley and Sons.

Recently developed prototypes of electro-adhesion pads include an aluminized mylar clamp [47], a stamp-printed carbon electroded silicone membrane [49] and sputtered copper-electroded polyimide film [48]. The fabrication method varies with electrode size and material. Stamp printing is good for mass production of carbon-based electrodes over a moderate area. Photolithography and the lift-off process are good for making high-resolution metallic electrodes over a small area. On the other hand, inkjet printing is scalable to print electrode from a small area to a large area. Recently, inkjet printing was used to print a pair of silver interdigital electrodes on a flexible omniphobic dielectric layer (a fluoroalkylated tracing paper). As shown in Figure 11, the printed silver interdigital electrodes have a finger length of 50 mm, a finger width of 0.5 mm and a finger gap of 0.5 mm over a large span of 115 mm. This large-areal (50 mm by 115 mm) printed electro-adhesion pad can support a 500-gram weight upon 2-kV activation across the interdigital electrodes.

### 4.9. Piezoelectric Polymer Actuators

Piezoelectric actuators are often used as a diaphragm pump to drive a microfluidic system. Conventional piezoelectric diaphragm actuators are made of piezoelectric ceramic screen printed and fired on a metal sheet, as for a piezoelectric beeper [28,30]. Such piezoelectric ceramic unimorphs of a few millimeters are however difficult to embed as an integral or monolithic part of a micro-fluidic system.

**Figure 12:**
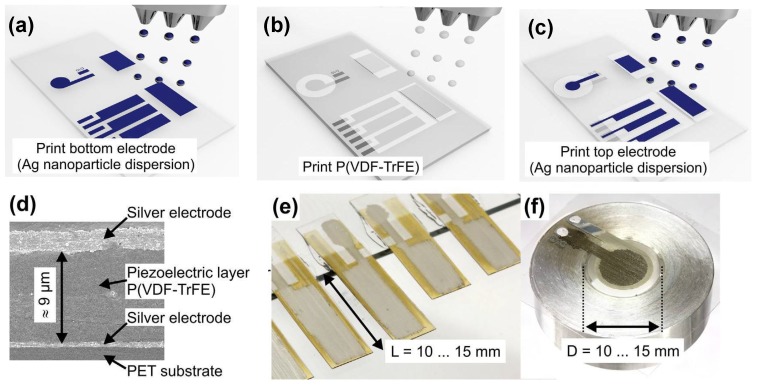
All inkjet-printed piezoelectric polymer actuator : (**a**–**c**) process steps for printing the bottom electrode, piezoelectric (PVDF-TrFE) layer, and top electrodes (sintering and tempering step not shown); (**d**) cross-section of the electroded PVDF layer; (**e**) piezoelectric cantilever; (**f**) a circular piezoelectric membrane on a glass plate with a hole. Adapted from [19], Copyright (2013), with permission from Elsevier.

Recently, researchers at Jena [19,50] showed an all-inkjet-printed piezoelectric polymer bi-layer membrane good for micropumping at a flow rate of several μL/min. Such a piezoelectric polymer actuator (15 mm long or in diameter) on a 125 μm-thick polyethylene terephthalate (PET) substrate is made of a polymer piezoelectric layer of PVDF-TrFE sandwiched between silver electrodes (see Figure 12). The PVDF-TrFE layer of 9 μm in thickness is a result of 100-layer inkjet printing from the solution of PVDF-TrFE in cyclopentanone and subsequent annealing at 130 ${}^{\circ }$C for 24 h that helps remove the solvent and increase the crystallinity of the β-phase PVDF. The top and bottom silver electrodes are inkjet printed from silver nanoparticle dispersion, which is subjected to post-printing sintering at 100${}^{\circ }$ for 1 h in a low-pressure argon plasma. A 15mm long cantilever of piezoelectric polymer/PET unimorph can bend a maximum deflection up to 145 μm at 400 V, showing a piezoelectric coefficient $d_{31}$ = 7–9 pm/V.

### 4.10. Gas and Humidity Sensors

Ambient or environmental conditions affect the well-being of plants, animals and humans. The parameters for environmental monitoring include temperature, humidity, gases and particles. Inkjet printing methods allow cost-effective fabrication of distributed gas and humidity sensors on a flexible substrate for environmental monitoring.

Gas sensors can measure the change of conductance across a functional layer in response to the presence of a target gas. Humidity sensors measure the capacitance change of a functionalized capacitor with interdigital electrodes. For example, the group at Ecole polytechnique federale de Lausanne (EPFL) has developed: (1) gas sensors based on a functional layer of tin oxide [51]; and (2) humidity sensors based on a functional layer of cellulose acetate butyrate (CAB) [16,52,53]. Such inkjet-printed gas sensors can be integrated with inkjet-printed radio frequency identification (RFID) for wireless communication [54].

**Figure 13:**
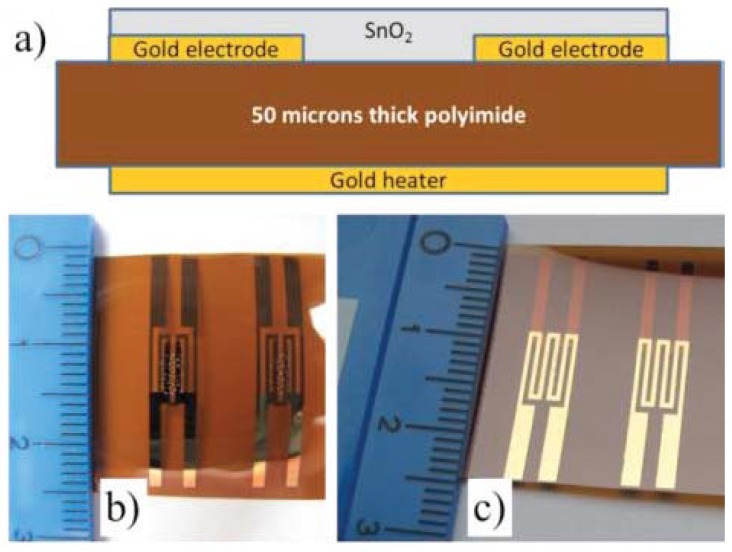
Inkjet printed gas sensor with a SnO${}_{2}$ functional layer, gold electrodes and a heater: (**a**) schematic showing the sensor section; (**b**,**c**) photographs in the top and angled views. Reproduced from [51], Copyright (2015), with permission from Elsevier.

**Figure 14:**
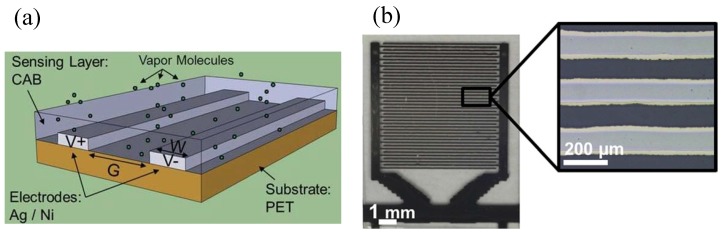
Inkjet A printed humidity sensor: (**a**) a schematic of two opposite electrodes with a polymeric sensing layer ; (**b**) an optical micrograph of a capacitor with inter-digital electrodes with an inset showing a zoom-in view . Reproduced from [52], Copyright (2012), with permission from Elsevier.

The gas or humidity sensors can be all inkjet printed. Two gold electrodes and a heater of the sensors are printed from either gold ink (Harima NPG-3) or silver ink with a line width of 100–150 μm. In a gas sensor (see Figure 13) to detect the presence of NO${}_{2}$ and CO [51], a functional layer of tin oxide between electrodes was printed from ethoxide tin sol-gel, which was later sintered in air at 400 ${}^{\circ }$C for an hour. Similarly, in a humidity sensor (see Figure 14) [52,53], a functional layer of cellulose acetate butyrate is printed from dissolved polymer, a solution of CAB in hexyl acetate. Inkjet printing of these functional materials allows large area deposition with adequately good lateral and thickness control.

### 4.11. Photo-Detector for Irradiation

**Figure 15:**
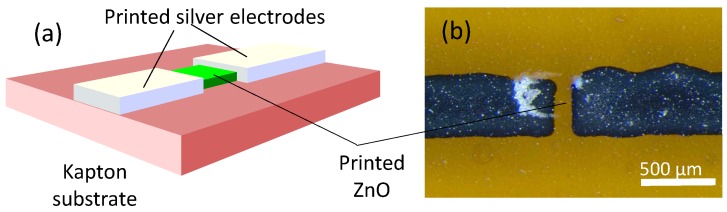
ZnO UV sensor: (**a**) schematic; (**b**) photograph. Redrawn with kind permission from Mr. Tran, V. T.

**Figure 16:**
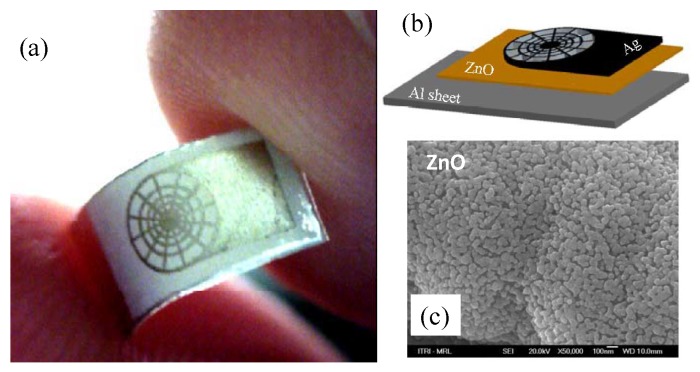
ZnO infrared sensor : (**a**) photograph of a complete device; (**b**) schematics showing the component layers; (**c**) a scanning electron micrograph showing the ZnO coating obtained from the inkjet printing and sintering processes. Reproduced from [29]. CC licenses (2010), MPDI Sensor.

Printed ZnO thin film was used to make a UV photo-detector [20], which is configured as a photoconductor between two printed silver electrodes (see Figure 15). Upon high-intensity UV illumination, this ZnO photo-detector generates a photocurrent. On the other hand, printed ZnO thin film also makes a pyroelectric sensor [29] (see Figure 16) as a dielectric sandwiched between a bottom aluminum electrode and a top web-shaped electrode of printed silver. Such a pyroelectric sensor can generate an output voltage upon heat absorption from an infrared source.

ZnO thin film can be printed from sol-gel. Typically, a precursor solution of ZnO is prepared by dissolving a zinc acetate in ethanol (magnetically stirred at 60 ${}^{\circ }$C for 1 h) [20] or ethylene glycol (stirred at 140 ${}^{\circ }$C for 0.5 h) [29]. After filtering, this particle-free precursor solution is suitable for inkjet printing. A substrate heating is needed to evaporate the solvent, while sintering at 400–500 ${}^{\circ }$C is needed to improve the piezoelectric or pyroelectric effect of printed ZnO thin film. This high sintering temperature of up to 500 ${}^{\circ }$C [29] may preclude the use of plastic substrate. The dot obtained from the printed and sintered ZnO sol-gel is subject to the coffee-ring effect [20], with a peak rim ranging from 100 nm–400 nm thick, due to the non-uniform temperature of the droplet upon contact with the hot substrate.

## 5. Potential Applications

**Figure 17:**
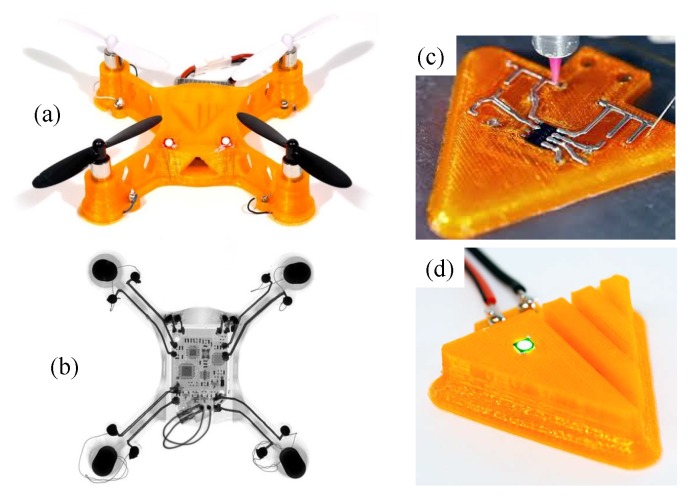
3D-printed quadcopter body with integrated silver wiring [56,57]: (**a**) a complete prototype; (**b**) x-ray image showing the wiring and embedded electronics; (**c**) printing of silver ink for wiring to a discrete electronic chip; (**d**) plastic encapsulation of silver wires and electronics. Reproduced with permission from Voxel8, Inc.

Parallel to inkjet printed transducers, 3D electronic printers have been recently developed to print a 3D printed circuit board, e.g., a quadcopter body with an integrated circuit [55]. Such 3D electronic printers (e.g., Voxel8 [56,57]) combine a fused filament fabrication print head for thermoplastics or UV resin with a conductive silver ink print head (see Figure 17). These 3D printing techniques will be useful for rapid prototyping of bio-inspired thoracic compliant mechanisms [58] for flapping wing micro air vehicles.

Hybrid integration of inkjet printed transducer arrays can add sensory and additional function to the 3D-printed mobile robot. It is foreseen that electro-adhesion pad can be integrated with the drone to enable perching onto a branch like a bat does [59]. Whereas, the electro-adhesion pads have been used to enable wall climbing of mobile robot [47]. Integration of gas sensors [51,53] with bioinspired drones or mobile robots can also open the possibility of environmental surveillance.

## 6. Challenges and Prospects

This review shows that inkjet printing is good for printing two-dimensional or surface MEMS devices from a small unit to an array over a large area, while multiple printing can make three-dimensional (3D) sensors and actuators. Looking ahead, there are ample rooms and possibilities for inkjet printing techniques to realize multifunctional areal transducers.

Though the printed conductor is nearly as good as the bulk conductor, current printable dielectric material does not have high dielectric strength. Recent development of aerosol jet printers, e.g., Optomec [55,60], shows high resolution printing of conductor or dielectric is possible. This will hopefully eliminate the need for other fabrication methods, like spin coating or chemical vapor deposition, to make high quality dielectrics or conductors.

So far, the inkjet printing method alone does not make many multi-functional MEMS like silicon micro-machined ones. Inkjet printing of multi-materials will be a rewarding challenge to enrich the functions MEMS, just like multi-material MEMS on silicon wafer [1,2,61]. With more print heads and printable materials, which ranges from UV curable elastomer, plastic, metal to dielectric, it is not far from now to fully print functional sensors and actuators [62,63,64]. In addition, the improvement in the print resolution and removal resolution could help rapid prototyping of accurate sensors and actuators.
